# Laryngeal Squamous Cell Carcinoma Is Characterized by a Stronger Expression of Nectin-4 Compared to Nectin-2

**DOI:** 10.3390/cimb47050296

**Published:** 2025-04-23

**Authors:** Matej Maršić, Nives Jonjić, Maja Gligora Marković, Svjetlana Janković, Marko Velepič, Ilinko Vrebac, Lara Batičić, Tamara Braut

**Affiliations:** 1 Clinical Hospital Centre Rijeka, Otorhinolaryngology and Head and Neck Surgery, 51000 Rijeka, Croatia; marsic.matej@gmail.com (M.M.); marko.velepic123@gmail.com (M.V.); ilinko.vrebac@gmail.com (I.V.); 2 Clinical Hospital Centre Rijeka, Pathology and Cytology, 51000 Rijeka, Croatia; nives.jonjic@medri.uniri.hr; 3 Department of Biomedical Informatics, Faculty of Medicine, University of Rijeka, 51000 Rijeka, Croatia; majagm@medri.uniri.hr; 4 Faculty of Medicine, University of Rijeka, 51000 Rijeka, Croatia; sjankovic@uniri.hr; 5 Department of Medical Chemistry, Biochemistry and Clinical Chemistry, Faculty of Medicine, University of Rijeka, 51000 Rijeka, Croatia

**Keywords:** nectin-2, nectin-4, laryngeal squamous cell carcinoma (LSCC), tissue microarray (TMA), immunohistochemistry (IHC), cell adhesion molecules, biomarkers, head and neck squamous cell carcinoma (HNSCC)

## Abstract

Nectin-2 and Nectin-4 are cell adhesion molecules associated with the progression of various cancers. The main goal of this pilot study was to evaluate the expression patterns of Nectin-2 and Nectin-4 in laryngeal squamous cell carcinoma (LSCC). A retrospective study was conducted on tissue microarray (TMA) samples derived from 31 patients who underwent total laryngectomy. The findings revealed heterogenous expression of both Nectin-2 and Nectin-4 in tumor cells and surrounding stroma, with Nectin-4 expression being significantly higher than Nectin-2 expression. Specifically, 74% of cases showed weak cytoplasmic staining for Nectin-2, while 41.93% exhibited strong cytoplasmic staining for Nectin-4. Both Nectin-2 and Nectin-4 expressions were more pronounced at the invasive tumor margins. Although no significant differences in Nectin-4 expression were observed across tumor grades (W = 83.500; z = −0.463; *p* = 0.658), differences in expression patterns were noted. Well-differentiated tumors (Grade 1), 80.65% of cases, showed predominantly membranous Nectin-4 staining, including in squamous epithelial cells of the mucosal surface. Conversely, in less-differentiated tumors (Grade 2 and 3), a shift toward cytoplasmic staining was evident. Specifically, 74.19% of Grade 2 tumors and 100% of Grade 3 tumors showed a predominant cytoplasmic localization of Nectin-4. This transition from membranous to cytoplasmic localization was also evident in the progression from normal superficial epithelium to malignant tissue. These observations suggest that alterations in the expression and subcellular localization of Nectin-4 may be associated with carcinogenesis and could serve as potential markers for the assessment of precancerous lesions and the aggressiveness of laryngeal tumors.

## 1. Introduction

Laryngeal squamous cell carcinoma (LSCC) accounts for over 95% of malignant laryngeal tumors, requiring intensive, multidisciplinary, and multimodal treatment strategies, which unfortunately often result in unsatisfactory outcomes [1]. Epidemiological data show that LSCC has an incidence of 2.76 cases, a prevalence of 14.33 cases, and a mortality rate of 1.66 deaths per 100,000 people annually [2], e.g., data from 2020 reported 184,615 new cases globally, with approximately 470,000 individuals living with the disease within five years of diagnosis and 99,840 related deaths. In Europe, 39,899 new cases and a 5-year prevalence of 115,000 were recorded. In Croatia, approximately 300 new cases occur annually, with a 5-year prevalence of 918 and approximately 150 deaths per year, reflecting a relatively high mortality rate. From 2013 to 2019, the 5-year survival rate was 61.6%, emphasizing the need for advancements in diagnostic and therapeutic approaches [3,4].

LSCC arises from the epithelial lining of the laryngeal mucosa and is primarily linked to prolonged exposure to carcinogens. Major etiological factors include chronic tobacco use, excessive alcohol consumption, and occupational hazards such as asbestos exposure [5]. In younger patients, HPV, particularly type 16, has emerged as a significant contributing factor to LSCC development [6,7,8,9,10]. LSCC is a multifactorial disease, in which genetic susceptibility, gastroesophageal reflux disease (GERD), and nutritional deficiencies (e.g., vitamin A) may also contribute to its pathogenesis [11].

The prognosis of LSCC is closely linked to early diagnosis and timely therapeutic intervention. Molecular biomarkers have emerged as critical tools for enhancing diagnostic precision, predicting tumor progression, and guiding personalized therapeutic interventions [12]. Among these, cell adhesion molecules such as Nectin-2 (PVRL2) and Nectin-4 (PVRL4) have emerged as potential molecular candidates due to their roles in tumorigenesis, epithelial integrity, and immune modulation as tumor-specific ligands for the immune checkpoint receptor TIGIT [13,14]. High expressions of Nectin-2 and Nectin-4 have been linked to tumor invasion, angiogenesis, and immune evasion in various epithelial cancers, indicating their potential as markers for tumor aggressiveness and early detection of premalignant lesions [15].

Nectins and Nectin-like molecules (Necls) are members of the immunoglobulin-like (Ig-like) transmembrane cell adhesion molecule (CAM) family, involved in mediating calcium-independent intercellular adhesion and regulating key cellular processes, such as motility, proliferation, differentiation, polarization, and apoptosis [16]. The high expression of Nectin-2 in breast and ovarian cancer [17,18], gallbladder cancer [19], and esophageal squamous cell carcinoma [20] has an impact on the invasiveness and aggressiveness of the malignant process. In pancreatic adenocarcinoma, tumor-associated neutrophils (TANs) upregulate Nectin-2, which contributes to tumor progression, promoting CD8^+^ T-cell exhaustion and facilitating an immunosuppressive tumor microenvironment [21]. Findings about Nectin-2 expression in lung adenocarcinoma suggest that it may be a potential target for immunotherapy [22], and tumors located in the right colon were found to have a higher likelihood of expressing Nectin-2, along with other Nectins, compared to those in the left colon, highlighting the potential prognostic value of Nectins in colorectal cancer [23].

Extensive research has demonstrated the overexpression of Nectin-4 across a variety of malignant tumors, including breast cancer [24,25], ovarian carcinoma [26], hepatocellular carcinoma [27], gastric cancer [28], pancreatic cancer [29], bladder cancer [30,31,32], cutaneous squamous cell carcinoma [33,34], esophageal cancer [35], and salivary gland cancer [36]. The study conducted by Dekanić A. et al. found that Nectin-2 and Nectin-4 may independently indicate prognosis in Grade 2/3 glioma patients [37]. In many of these malignancies, high levels of Nectin-4 correlate with increased tumor aggressiveness and poorer clinical outcomes. However, in breast cancer, Nectin-4 expression has been associated with better survival rates, likely due to its effectiveness as a therapeutic target [38].

In the study of Sanders et al. on head and neck squamous cell carcinoma (HNSCC), Nectin-4 is expressed in approximately 86.2% of cases, with moderate to high expression in 32.7% and pronounced expression levels found in non-smokers and p16-positive patients, and its presence has been linked to improved overall survival, suggesting its potential as a prognostic biomarker and a therapeutic target [39].

Despite the emerging data on Nectin-4 in HNSCC, its specific role in LSCC is underexplored. As HNSCC is a highly heterogeneous group of tumors with significant differences in histology and prognosis depending on the anatomical site, studying each subsite, such as the larynx, individually is essential. Currently, no published studies have specifically investigated Nectin-4 in the context of LSCC carcinogenesis. Extending previous work at our clinic involving immunohistochemical analysis of biomarkers like EGFR, IMP3, TGF-α, Ki-67, cyclin D1, and p53 [40,41,42,43,44,45,46], we aimed to expand our biomarker research by focusing on Nectin-2 and Nectin-4. The objective of this pilot study was to investigate and evaluate the expression of Nectin-2 and Nectin-4 in LSCC using tissue microarrays (TMAs) and immunohistochemistry (IHC) and to provide a more comprehensive understanding of their role in tumor biology, their aggressiveness and metastatic behavior, and the overall prognostic value of these molecules. We hypothesized that this analysis would enhance the knowledge about Nectin-2 and Nectin-4 regarding their potential function as novel biomarkers associated with tumor aggressiveness and metastatic potential in LSCC, contributing to risk stratification and therapeutic decision-making.

## 2. Materials and Methods

### 2.1. Tumor Samples

All the patients included in this retrospective study were diagnosed with LSCC and underwent total laryngectomy at the Otorhinolaryngology and Head and Neck Surgery of the Clinical Hospital Center Rijeka. Histopathological analysis of surgically obtained tissue was performed at the Clinical Department of Pathology and Cytology of the Clinical Hospital Center Rijeka and the Faculty of Medicine, University of Rijeka, from 2015 to 2024. In total, 31 paraffin samples of the larynx were collected for IHC analysis of Nectin-2 and Nectin-4.

### 2.2. Tissue Microarray Construction and Immunohistochemistry

Tissue microarrays (TMAs) were constructed using triplicates of 2 mm cores obtained from formalin-fixed, paraffin-embedded (FFPE) LSCC specimens (*n* = 31). The TMA blocks were assembled manually at the Department of Clinical Pathology and Cytology of the Clinical Hospital Center Rijeka, utilizing a manual tissue microarrayer (Alphelys, Plaisir, France). Normal liver tissue was included in the block layout to serve as a histological orientation marker. Cores were arranged at 0.5 mm intervals along both the x- and y-axes to ensure uniform spacing. From each TMA block, 4 µm thick sections were cut. One of the sections was stained with hematoxylin and eosin for morphological validation, while serial sections were prepared for immunohistochemical (IHC) staining. These sections were then deparaffinized using xylene substitute and rehydrated through a graded alcohol series.

Antigen retrieval for Nectin-2 was carried out by treating tissue sections with Tris/EDTA buffer (pH 9.0) for 15 min in a water bath. For Nectin-4, antigen retrieval was performed by immersing the sections via EnVision FLEX Target Retrieval Solution, with a high pH (3 in 1), and treating them for 20 min using a PT-Link instrument.

Subsequent immunohistochemical procedures were carried out using an automated immunostainer, the Dako Autostainer Plus system (manufacturer DakoCytomation, Fort Collins, CO, SAD, USA), following the manufacturer’s protocol, and using DakoREAL solutions (Dako, Glostrup, Denmark). For visualization of Nectin-2 and Nectin-4, primary antibodies were used, as shown in Table 1. Visualization of IHC staining was achieved with the EnVision Flex+ system (K8000; Dako, Glostrup, Denmark) with 3,3′-diaminobenzidine (DAB) as the chromogenic substrate, and slides were counterstained with Mayer’s hematoxylin. For negative controls, the primary antibody was substituted with phosphate buffered saline (PBS).

Immunostaining was evaluated semi-quantitatively using an Olympus BX46 light microscope (Olympus Corporation, Tokyo, Japan) under 200× magnification. Immunoreactivity of Nectin-2 and Nectin-4 was observed in tumor cells, with staining localized to the membranous and/or cytoplasmic compartments.

### 2.3. Evaluation of Staining

To assess the immunoreactivity of Nectin-2 and Nectin-4, both the percentage of positively stained tumor cells and the staining intensity were evaluated for each case. The percentage of positive cells was estimated in relation to the total tumor cells across the two examined cores. Staining intensity was classified into 4 grades (Table 2).

A histological score (H-score), ranging from 0 to 300, was calculated for each sample using the following formula: H-score = (1 × % weakly stained cells) + (2 × % moderately stained cells) + (3 × % strongly stained cells). The staining assessments were independently conducted by two experienced observers (MM and NJ), both blinded to the patient’s clinical data. In instances where discrepancies in staining intensity were observed, the two observers reexamined and reevaluated the slides together and reached a consensus for these cases.

### 2.4. Statistical Analysis

In data processing, the tool Excel was used for the data collection and entry required for further statistical analysis. In our data analysis, we used JASP software (version 0.19.1; JASP Team, Amsterdam, the Netherlands), and appropriate methods were used to test data distribution, such as the Shapiro–Wilk test and the Wilcoxon test or the Paired Sample *t*-test for examining differences between variables. The Wilcoxon test was conducted to assess the difference between the variables of cytoplasmatic (C) and membranous (M) H-score *of* Nectin-2 and H-score of Nectin-4 considering that these are two dependent groups of subjects and the collected data do not follow a normal distribution.

## 3. Results

This retrospective study aimed to better identify the pattern of expression of two adhesion molecules from the family of Nectins in LSCC. Archived tissue samples from 31 patients were retrieved from the database, and histopathological analysis of surgically obtained tissues was conducted at the Clinical Department of Pathology and Cytology, Clinical Hospital Center Rijeka, and the Faculty of Medicine, University of Rijeka. Nectin-2 and Nectin-4 expression levels were evaluated using immunohistochemical (IHC) analysis on tissue microarrays (TMAs) in LSCC tissues. The characteristics of patients with LSCC are listed in Table 3.

One of the goals of our work was to determine whether Nectin-2 and Nectin-4 are expressed in LSCC. The IHC staining results for Nectin-2 and Nectin-4 in LSCC confirmed the expression of both molecules in the tumor parenchyma and the surrounding stroma. Initial staining revealed distinct differences in their expression patterns. Nectin-4 exhibited strong expression in tumor cells, as well as in glandular epithelium and endothelial cells. In contrast, Nectin-2 showed weaker expression in both tumor cells and the stroma, with the exception of stronger staining observed in follicular dendritic cells within secondary lymphoid follicles.

**Figure 1:**
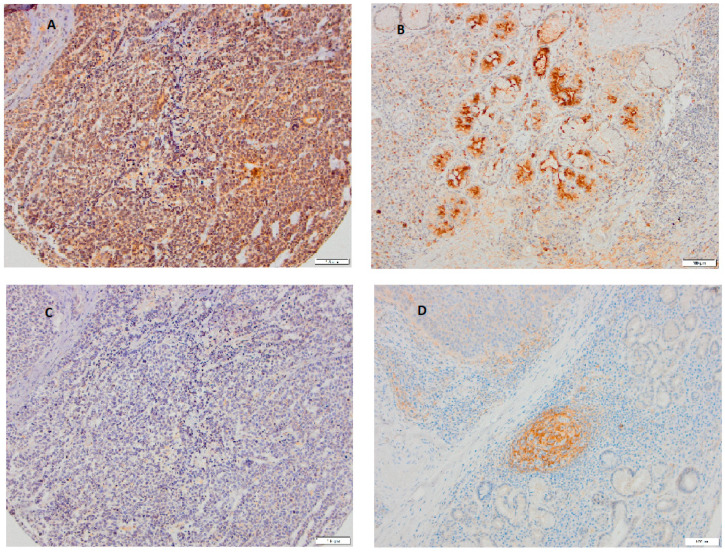
A representative case of poorly differentiated LSCC showing typical immunohistochemical staining of Nectin-2 and Nectin-4 on parenchymal and stromal tumor cells. Tumor cells (**A**) and glandular tissue (**B**) show that strong expression of Nectin-4 while Nectin-2 is almost negative on tumor cells (**C**) but positive on follicular dendritic cells in secondary lymphoid follicles (**D**), ((**A**–**D**): magnification 100×, scale bar 100 μm).

Figure 1 shows a representative case of poorly differentiated LSCC in which there is a notable difference in the expression of these two adhesion molecules both on parenchymal tumor cells and in the stromal cells.

**Figure 2:**
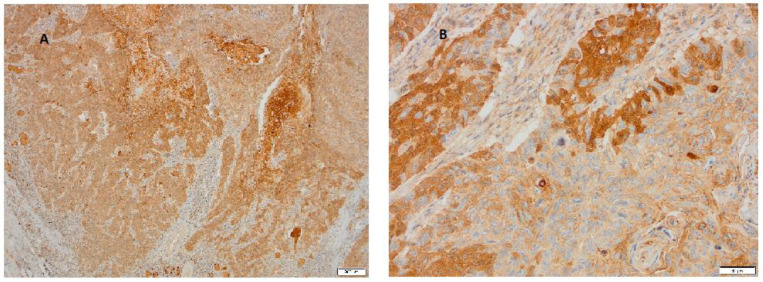
Representative case of LSCC immunohistochemically stained for Nectin-4. (**A**) Tumor cells show heterogenous cytoplasmic expression in a range from weak and moderate to very strong expression (magnification 100×, scale bar 200 μm). (**B**) At higher magnification, in addition to heterogenous cytoplasmic staining, focally strong membranous staining is visible (magnification 200×, scale bar 50 μm).

Our next goal was to determine the site of expression of these two adhesion molecules in the cytoplasmic and in the membranous parts of the tumor and to describe appearance patterns as more or less heterogeneous. IHC staining showed that heterogeneity in the expression of both Nectins is found within the tumor itself but that Nectin-4 expression is much stronger. Figure 2 shows heterogenous cytoplasmic staining for Nectin-4.

**Figure 3:**
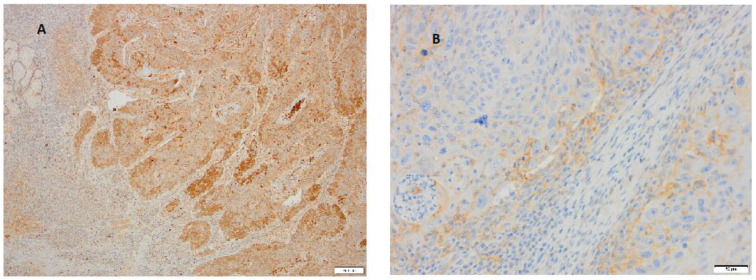
Representative cases of LSCC immunohistochemically stained for Nectin-2 and Nectin-4 at tumor margins. (**A**) In addition to heterogenous expression, which is more pronounced with Nectin-4, the image shows stronger staining of Nectin-4 in cells at periphery of tumor (magnification 40×, scale bar 200 μm). (**B**) At the higher magnification at the invasive edges of the carcinoma tumor, cells are positive for Nectin-2 also (magnification 200×, scale bar 50 μm).

In addition to the observed cytoplasmic staining of cancer tissue predominantly with the adhesion molecule Nectin-4, in our further research, we determined stronger expression of both Nectins at the invasive edges of the tumor, although it was much weaker with Nectin-2 (Figure 3).

After observing the expression of Nectin-2 and Nectin-4 in different tumor sites (membrane, cytoplasm, tumor edges) and regarding the heterogeneity of tissues after IHC staining, our next aim was to statistically analyze whether the difference in cytoplasmic and membrane expression between Nectin-2 and Nectin-4 is significant or not.

For that purpose, the Wilcoxon test was performed to evaluate the difference between the H-scores for Nectin-2 and Nectin-4 given that these variables represent two dependent groups (*n* = 31) and the data do not follow a normal distribution. The analysis revealed a statistically significant difference in cytoplasmic samples of LSCC between the H-score for Nectin-2 and H-score for Nectin-4 (W = 0.000, z = −4.703, *p* < 0.001). This statistical significance confirms a genuine difference in the distribution of values between the two markers in LSCC cytoplasmic samples, as presented in Table 4.

The Wilcoxon test was also conducted to assess the difference between the variables of the H-scores for Nectin-2 and Nectin-4 in membranous LSCC. The results showed no statistically significant difference between the observed variables of the H-scores for Nectin-2 and H-score for Nectin-4 in membranous sample (W = 83.500; z = −0.463, *p* = 0.658), as presented in Table 5.

**Figure 4:**
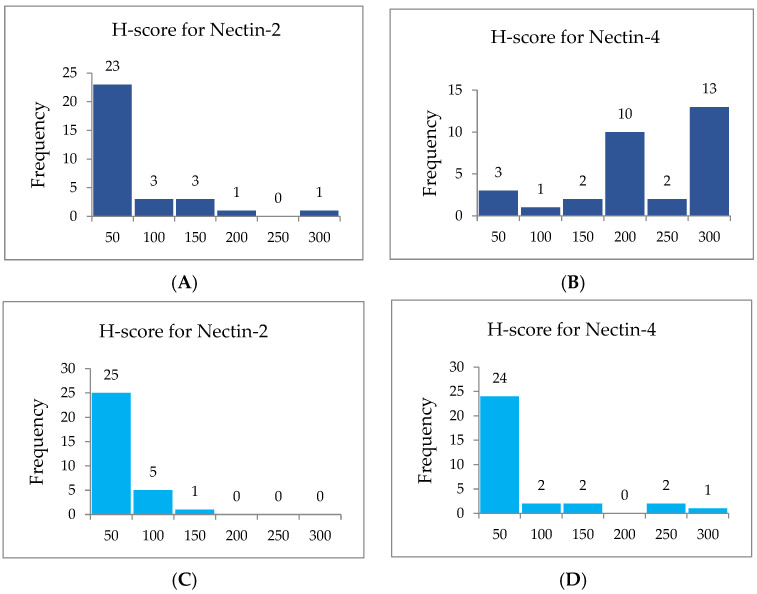
Distribution of patients with LSCC (frequency) according to cytoplasmic and membranous immunohistochemical H-scores in a range from the lowest to the highest intensity: 0–50 (lowest), 51–100, 101–150, 151–200, 201–250, 251–300 (highest). (**A**) Cytoplasmic immunohistochemical H-scores for Nectin-2. (**B**) Cytoplasmic immunohistochemical H-scores for Nectin-4. (**C**) Membranous immunohistochemical H-scores for Nectin-2. (**D**) Membranous immunohistochemical H-scores for Nectin-4.

The distribution of the cytoplasmic and membranous H-scores for Nectin-2 and Nectin-4 of patients (*n* = 31) with LSCC is clearly illustrated in Figure 4.

Figure 4 represents cytoplasmic (A, B) and membranous (C, D) H-scores for Nectin-2 and Nectin-4. A histoscore with a potential range of 0–300 that was calculated according to the intensity of the immunohistochemical reaction in the tissue sample as 0 (negative), 1 (weak), 2 (moderate), or 3 (strong) and then classified into six groups from lowest to highest intensity of histochemical tissue staining. IHC staining for Nectin-2 in the cytoplasm of LSCC samples for 23 patients (74.19%) out of total number of 31 patients showed weak staining in the range of 0–50. On the contrary, IHC staining for Nectin-4 in the cytoplasm of LSCC samples for 13 patients (41.93%) out of the total number of 31 patients showed strong staining in the range of 251–300 and for 10 patients (32.25%) out of the total number of patients moderate staining in the range of 151–200.

H-scores for Nectin-2 and Nectin-4 in each cytoplasmic laryngeal tumor sample (*n* = 31) are listed in Table 6.

H-scores for Nectin-2 and Nectin-4 in each membranous laryngeal tumor sample (*n* = 31) are listed in Table 7. IHC staining for Nectin-2 in the membrane of LSCC for 25 patients (80.64%) out of the total number of 31 patients showed weak staining in the range of 0–50. Likewise, IHC staining for Nectin-4 in the membrane of LSCC for 24 patients (77.41%) out of the total number of 31 patients showed weak staining in the range of 0–50, as presented in Table 7.

Our findings demonstrated a significantly stronger expression of Nectin-4 in LSCC, prompting further investigation into whether variations in Nectin-4 expression influence tumor differentiation. Due to the limited number of well-differentiated (Grade 1) and poorly differentiated (Grade 3) tumors in our study, comprehensive statistical analysis was not performed, with the analysis focusing primarily on the moderately differentiated tumors (Grade 2). Ongoing research with a larger sample size is expected to provide additional insights, and results from this expanded study will be published in subsequent reports.

Tumors staging and grading were based on criteria outlined in the WHO Classification of Head and Neck Tumors [47]. Among the 31 patients included in this study, 29 patients presented with T3 stage tumors and 2 patients with T4 stage tumors. Histologically, 20 patients presented with Grade 2 (G2) tumors, 6 patients had Grade 1 (G1), and 5 patients had Grade 3 (G3) LSCC. The median age of the patients was 66.6, with a mean age of 66.0 years (SD = 8.5). This study consisted predominantly of males, with 30 male patients (96.77%) and 1 female patient (3.22%).

**Figure 5:**
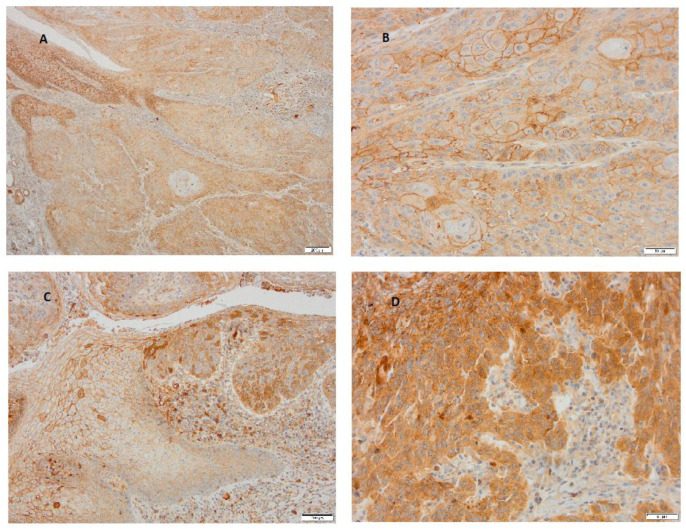
A representative case of well- and poorly differentiated LSCC stained immunohistochemically with Nectin-4. (**A**) Well-differentiated carcinoma with heterogenous expression of Nectin-4 is covered on the surface by a multilayered squamous epithelium of lamina epithelial mucosa, which shows strong membranous expression (magnification 40×, scale bar 200 μm). (**B**) At higher magnification of the same carcinoma, the membranous staining is also observed in squamous tumor cells (magnification 200×, scale bar 50 μm). (**C**) In the carcinoma itself, less-differentiated tumor cells express stronger cytoplasmic staining for Nectin-4 (magnification 200×, scale bar 50 μm). (**D**) At the edge of the poorly differentiated cells of LSCC, the transitional layer of cells gradually turn from normal stratified squamous epithelium into a neoplastic layer, and different expression levels of Nectin-4 from membranous to cytoplasmic layers are seen (magnification 100×, scale bar 100 μm).

The analysis of the immunohistochemical expression of Nectin-4 revealed distinct expression patterns between well-differentiated and poorly differentiated tumors. Well-differentiated tumors (Grade 1) exhibited predominantly membranous Nectin-4 staining, including in the squamous epithelial cells of the mucosal surface (Figure 5A,B). In contrast, less-differentiated tumors (Grade 2 and 3) showed a marked shift toward the cytoplasmic expression of Nectin-4 (Figure 5C,D). These observations indicate a potential association between Nectin-4 expression patterns and the degree of tumor differentiation, indicating the necessity for further investigation in larger studies.

## 4. Discussion

LSCC is a highly aggressive malignant form of head and neck cancer with significant implications for patient survival and quality of life. The prognosis of LSCC is closely tied to early detection and initiation of treatment, because early-stage tumors can often be effectively managed with surgery or radiation monotherapy, with a high probability of preserving the laryngeal structure and function. However, late-stage disease typically requires aggressive multimodality therapy and is associated with lower survival rates and reduced chances for organ preservation. Such interventions in the later stages usually result in long-term disability, affecting the quality of life of the patient and exerting a broader impact on the patient family and the healthcare system [48,49,50]. Along with these challenges, the need for identifying reliable molecular biomarkers that aid in early detection and therapeutic targeting is an important task in the translational oncology field.

To the best of our knowledge, this study represents the first systematic analysis of Nectin-2 and Nectin-4 expression in LSCC using IHC analysis on TMAs from surgical tissue specimens of patients who underwent total laryngectomy. The main objective was to evaluate the expression patterns of Nectin-2 and Nectin-4 within the tumor parenchyma and the surrounding stroma. Using IHC staining, we confirmed the expression of both biomarkers, with marked differences in expression of intensity and in distribution patterns. Nectin-4 demonstrated strong expression in tumor cells and glandular epithelium, whereas Nectin-2 exhibited overall weaker expression, primarily localized to the surrounding stromal tissue and follicular dendritic cells within secondary lymphoid follicles. A detailed analysis of subcellular localization revealed heterogeneity in both cytoplasmic and membranous staining across tumor regions. Nectin-4 displayed strong heterogeneous cytoplasmic staining and focally strong membranous staining in specific tumor areas.

Enhanced staining at the invasive tumor margins was observed for both Nectin-2 and Nectin-4, with a more pronounced pattern for Nectin-4, suggesting involvement of this molecule in tumor invasiveness and progression. We also wanted to quantify these observations by using H-scores calculating both cytoplasmic and membranous staining patterns. Cytoplasmic H-scores were statistically significantly higher for Nectin-4 compared to Nectin-2, while no significant difference was found in membranous H-scores. Pronounced cytoplasmic staining of Nectin-4 was observed in 41.94% of cases, whereas only 3.23% of samples showed the same level of expression for Nectin-2.

Furthermore, an important aspect of our study involved the analysis of correlation between Nectin-4 localization and tumor differentiation. We found that in well-differentiated tumors, Nectin-4 was predominantly membranous, particularly in squamous epithelial cells of the mucosal surface. Conversely, in less-differentiated tumors, a shift toward cytoplasmic localization was noted. These findings are consistent with the research literature indicating the oncogenic role of Nectin-4 across multiple cancer types. The research of Deng et al. in esophageal cancer found high Nectin-4 expression both in the cytoplasm and in the membrane of the tumor cells, with higher expression correlating with larger tumor size, advanced stage, and reduced overall survival, and the same distribution in pancreatic cancer was found, indicating that high expression was associated with poor postoperative prognosis, with median survival times of 426 days for patients whose samples showed a high expression of Nectin-4 compared to 682 days for patients whose samples show a low expression of Nectin-4, while in hepatocellular carcinoma, Nectin-4 expression was predominantly cytoplasmic, with higher staining intensity observed in cases from metastatic and progressive tissue samples, and these findings further indicate the involvement of Nectin-4 in tumor progression [29,35,51].

Our findings, supported by existing research, reinforce the importance of Nectin-4 in cancer biology. Strong cytoplasmic expression, especially in invasive regions of tumor tissue and in poorly differentiated tumors, as well as its correlation with advanced disease stages in various malignancies, highlights its potential utility as a prognostic biomarker and therapeutic target for LSCC. These findings suggest that Nectin-4 overexpression and predominantly cytoplasmic accumulation may serve as indicators of tumor aggressiveness and differentiation in LSCC. The observed shift from the membranous to the cytoplasmic region in Nectin-4 localization could represent a surrogate marker of malignant transformation and invasiveness, potentially aiding in the identification of premalignant or dysplastic lesions in the early stage of disease. As Nectin-4 is a member of the immunoglobulin-like cell adhesion molecule family, its role is implicated in tumorigenesis through its effects in cell motility, invasion, and metastasis. Its consistent overexpression in LSCC, particularly at tumor margins and in less-differentiated tumors, further supports its biological significance.

Contradictory findings have been reported regarding the prognostic significance of Nectin-4 in HNSCC. While most studies associate Nectin-4 expression with poorer prognosis, some have reported the opposite. For example, Sanders et al. observed longer survival in patients with Nectin-4-positive tumors [39]. Similar findings were noted by Tanaka et al. in cases of cutaneous SCC [34]. These discrepancies may be attributable to variations in methodology, tumor scoring systems, or sample selection. It is also important to consider that HNSCC encompasses a highly heterogeneous group of tumors, differing in histology, biological behavior, and prognosis. Therefore, further research is needed to clarify the prognostic value of Nectin expression within specific anatomical subtypes. Our study focuses on LSCC, providing new insights into the roles of Nectin-2 and Nectin-4—both of which remain relatively underexplored in this particular cancer type.

To our knowledge, the only large-scale study was conducted by Sanders et al. [39]. They evaluated Nectin-4 expression in 159 HNSCC tissue samples (from the oral cavity—7 cases; from the oropharynx—59 cases; from the hypopharynx—7 cases; from the larynx—28 cases; and 49 unknown cases) using the IHC method. The study revealed that Nectin-4 was expressed in 86.2% of HNSCC cases, moderate to high expression was found in 32.7% of cases, and normal mucosa showed low to moderate expression in 96.4% of patients [39]. Similar to findings in our study, which are more detailed, Nectin-4 localization included both cytoplasmic and membranous staining. The importance of our study lies in its focus on laryngeal cancer in contrast to previous research that examined a broad spectrum of head and neck tumors, which vary significantly in prognosis depending on their anatomical location. The study of Sanders et al. highlights the potential of Nectin-4 as both a prognostic marker and a therapeutic target and also raises the possibility of Enfortumab Vedotin (EV), an antibody–drug conjugate targeting Nectin-4, being explored in future trials for selected HNSCC cases. The antibody–drug conjugate EV is FDA-approved for metastatic urothelial carcinoma [52,53] and is under investigation in other malignancies, including squamous cell carcinomas of the breast and skin, with ongoing trials in combination with immune checkpoint inhibitors (ICIs) in different cancers further underlining its relevance in oncology [16,24,25,26,33,54,55,56,57,58]. A Phase II clinical trial evaluating EV in head and neck squamous cell carcinoma (HNSCC) is currently underway, underscoring the growing interest in targeting Nectin-4 in this region [59].

Our data indicate a possible correlation between cytoplasmic Nectin-4 expression and tumor differentiation and are aligned with the study of Mayer et al. on salivary gland tumors. They found that membranous Nectin-4 expression was seen in 30.3% of cases of primary tumors and 55.0% of cases of lymph node metastases, while cytoplasmatic expression comprised 77.0% of primary tumors and 80.0% of lymph node metastases, confirming correlations between Nectin-4 expression and histopathological features like high tumor grade, lymph node metastasis, and advanced stage of tumor development [60].

In conclusion, our study provides the first detailed analysis of Nectin-2 and Nectin-4 expression in LSCC. Our results demonstrate significantly higher and more heterogenous Nectin-4 expression, especially in the cytoplasm of the tumor samples, and indicate that Nectin-4 may serve as a diagnostic, prognostic, and therapeutic biomarker in LSCC.

## 5. Conclusions

To the best of our knowledge, this is the first immunohistochemical study to evaluate the expression of Nectin-2 and Nectin-4 in LSCC. Our findings demonstrate a significantly higher expression of Nectin-4 in tumor cells compared to Nectin-2. Notably, Nectin-4 overexpression was predominantly cytoplasmic in poorly differentiated tumors, whereas membranous staining was more pronounced in well-differentiated carcinomas and in the cell membranes of normal epithelial tissue. This observed shift from membranous to cytoplasmic localization of Nectin-4 in tumor cells may reflect its involvement in the process of laryngeal carcinogenesis and may represent the basis for further important research in this context. With further validation in larger studies, Nectin-4 could be integrated into biomarker panels for early detection and treatment stratification and may also represent a promising target for molecular therapies in different stages of LSCC.

## Figures and Tables

**Table 1:** Immunostaining with primary antibodies used for immunohistochemical detection of Nectin-2 and Nectin-4.

Target Protein	Antibody Type Used	Clone	Manufacturer	Dilution	Diluent
Nectin-2	rabbit monoclonal (recombinant) antibody	EPR21124/ab233384	Abcam (Cambridge, UK)	1:250	DAKO Antibody Diluent S0809
Nectin-4	rabbit polyclonalantibody	ab155692	Abcam	1:100	DAKO Antibody Diluent S0809

**Table 2:** Staining intensity classification.

0	Negative (no detectable staining at high magnification)
1	Weak (faint staining visible only at high magnification)
2	Moderate (easily visible at low magnification)
3	Strong (intense staining evident even at low magnification)

**Table 3:** Characteristics of patients with LSCC.

Varible	LSCC (*n* = 31)
Age	
Median age (years)	66.6
Range	49.1–87.4
Mean	66.0
Standard deviation	8.5
Gender	
Male	30 (96.77%)
Female	1 (3.23%)
T-stage	
T1	/
T2	/
T3	29 (93.55%)
T4	2 (6.45%)
SCC histologic grade	
Grade 1	6 (19.35%)
Grade 2	20 (64.52%)
Grade 3	5 (16.13%)

**Table 4:** Wilcoxon test for the variables of the cytoplasmic immunohistochemical H-scores for Nectin-2 and Nectin-4.

Measure 1	Measure 2	W	z	*p*	Hodges–Lehmann Estimate	Rank– Biserial Correlation	SE Rank– Biserial Correlation
H-score Nectin-2	H-score Nectin-4	0.000	−4.703	<0.001	−190.000	−1.000	0.209

**Table 5:** Wilcoxon test for the variables of the membranous immunohistochemical H-scores for Nectin-2 and Nectin-4.

Measure 1	Measure 2	W	z	*p*	Hodges–Lehmann Estimate	Rank– Biserial Correlation	SE Rank– Biserial Correlation
H-score Nectin-2	H-score Nectin-4	83.500	−0.463	0.658	−12.480	−0.121	0.256

**Table 6:** Frequencies of individual H-scores according to defined range for cytoplasmic expression of Nectin-2 and Nectin-4 (*n* = 31).

Cytoplasmic	H-Score for Nectin-2		H-Score for Nectin-4	
Range	Frequencies	Percentage	Frequencies	Percentage
0–50	23	74.19%	3	9.68%
51–100	3	9.68%	1	3.23%
101–150	3	9.68%	2	6.45%
151–200	/	/	10	32.26%
201–250	1	3.23%	2	6.45%
251–300	1	3.23%	13	41.94%
Total	31	100%	31	100%

**Table 7:** Frequencies of individual H-scores according to defined range for membranous expression of Nectin-2 and Nectin-4 (*n* = 31).

Membranous	H-Score for Nectin 2		H-Score for Nectin 4	
Range	Frequencies	Percentage	Frequencies	Percentage
0–50	25	80.65%	24	77.42%
51–100	5	16.13%	2	6.45%
101–150	1	3.23%	2	6.45%
151–200	/	/	/	/
201–250	/	/	2	6.45%
251–300	/	/	1	3.23%
Total	31	100%	31	100%

## Data Availability

The original contributions presented in this study are included in the article. Further inquiries can be directed to the corresponding authors.

## List of Abbreviations and Acronyms

ADC	Antibody–drug conjugate
EGFR	epidermal growth factor receptor
EV	enfortumab vedotin
HER-2	human epidermal growth factor receptor-2
Histo score	*H-score*
HNSCC	head and neck squamous cell carcinoma
IHC	immunohistochemistry
LSCC	laryngeal squamous cell carcinoma
TMA	tissue microarray
